# The effect of repeated porcelain firings on the marginal fit of millable and conventional casting alloys

**DOI:** 10.1371/journal.pone.0275374

**Published:** 2023-10-24

**Authors:** Rashin Giti, Mahdi Hosseinpour Aghaei, Farhad Mohammadi

**Affiliations:** 1 Department of Prosthodontics, School of Dentistry, Shiraz University of Medical Sciences, Shiraz, Fars, Iran; 2 Student Research Committee, School of Dentistry, Shiraz University of Medical Sciences, Shiraz, Fars, Iran; 3 Department of Pharmaceutics, School of Pharmacy, Shahid Sadoughi University of Medical Sciences and Health Care Services, Yazd, Iran; Semnan University, ISLAMIC REPUBLIC OF IRAN

## Abstract

The durability of dental restorations is highly determined by an accurate marginal fit, which is in turn affected by the high temperature of porcelain firing. Information is inadequate about the marginal adaptation of metal-ceramic restorations fabricated by soft metal milling technologies after repeated firings. This study aimed to compare the effect of repeated ceramic firings on the marginal fit of copings fabricated from cobalt-chromium through soft metal milling and a conventional nickel-chromium casting alloy. A single standard brass die was designed, machined, and scanned, based on which, 20 frameworks were designed and fabricated through either soft metal milling or conventional casting (*n* = 10 per group) and porcelain veneered. The vertical marginal fit of the metal copings was measured after 3, 5, and 7 firing cycles by using a digital microscope on 16 points around the finish line of the metal die at ×80 magnification. The data were analyzed through repeated measures ANOVA and independent t-test (*α* = 0.05). The marginal fit of neither metal group was significantly affected by the number of firing cycles (*P* = 0.747). However, the marginal discrepancy was statistically lower in the soft metal milling group than that in the casting group (*P*<0.001). Repeated porcelain firings did not significantly affect the marginal fit of either alloy and remained within the clinically acceptable range after firings. However, the milled alloy had superior marginal fit regardless of the number of firing cycles.

## Introduction

Metal-ceramic restorations have been widely used since 1960 due to their high mechanical strength and low cost [1, 2]. Their metal part is mainly made of dental casting alloys such as nickel-chromium (Ni-Cr) and cobalt-chromium (Co-Cr) [3]. Although Ni-Cr alloys are considerably rigid and elastic with high fusion temperature [4], they release ions during corrosion and have allergic potentiality [1, 5]. In comparison, Co-Cr alloys are more corrosion-resistant, have fewer side effects [1], and can be fabricated by various technologies like lost wax [6], milling [7], and selective laser melting [8].

The casting of base metal alloys through the traditional method of lost wax is a technique-sensitive procedure with difficult laboratory steps as the alloys have a high melting temperature and low ductility [6]. Several causes may contribute to an inaccurate marginal fit as impression material shrinkage, cast metal distortion, or wax patterns deformity [9]. Trying to omit the wax, computer-aided design/computer-aided manufacturing (CAD-CAM) has been introduced which uses the digital designation (CAD) to fabricate the framework through different production processes [10]. In the additive technique of selective laser melting, a laser scans a powder bed based on a CAD model and incrementally fabricates a geometrically free-form 3D object [11]. This method produces products of higher quality and reduces manufacturing time, human error, and material consumption [12].

Another CAD-CAM method was milling, which was first introduced to dentistry in the early 1970s [13–15]. Initially, frameworks were made of fully-sintered Co-Cr blanks [16], whose inherent hardness and low ductility made the milling difficult as it took more energy and damaged the milling tools like burs [2]. Attempts to solve such problems lead to the development of soft-milling which used pre-sintered Co-Cr blocks. In soft-milling, a pre-sintered polymer-bound block of powder Co-Cr is milled and sintered in an argon atmosphere to achieve full density [17]. It reduces the manufacturing time, costs, and stress applied to the milling machine [3].

Since the durability and efficacy of dental restorations highly rely on the marginal fit [18], lots of attempts have been made to minimize the gap between the tooth and restoration. Dissolution of cement over time creates a gap for plaque accumulation, which consequently causes inflammation of periodontal tissues, decay, increased gingival crevicular fluid, alveolar bone destruction, and treatment failure [19]. The acceptable value of marginal fit depends on the type of restoration, finish line design, cement type and viscosity, and relief of the crown inner surface [20, 21]. Although the clinically acceptable discrepancy value remains unclear, it should not exceed 120 μm [22].

Esthetic and clinical demands of metal-ceramic crowns are only achieved through multiple porcelain firings [23], whose high temperature can transform the metal framework. Besides, different thermal expansion coefficients of the alloy and ceramic can distort the restoration [8]. Few studies addressed the effect of repeated porcelain firing on the marginal fit of new metal-ceramic systems. Kocaağaoğlu et al. [1] evaluated the effects of three metal fabrication protocols (lost wax, soft milling, and laser sintering) and repeated ceramic firings (1, 2, and 3 simulated ceramic firing cycles) on the marginal fit of metal-ceramic restorations and found that ceramic firings did not significantly affect the marginal fit of their studied groups, but the lost wax protocol yielded restorations with significantly higher marginal discrepancy than those made through soft milling and laser sintering. In another study, Kaleli and Saraç [24] detected that the marginal adaptation in restorations made via direct powder-bed fusion was better than the lost wax and milling methods; and that porcelain application and cementation deteriorated the marginal discrepancy.

Porcelain almost always requires to be fired to achieve the appropriate morphology, occlusal relationship, contact with the adjacent teeth, and to obtain a clinically acceptable color for the metal-ceramic crowns. Therefore, the effect of this process on the marginal fit needs to be closely investigated. Due to the lack of enough study and the controversy between the studies, the present study was designed to evaluate the effect of multiple ceramic firings (3, 5, and 7) on the marginal fit of metal-ceramic crowns, the framework of which was made through CAD-CAM milling and conventional casting. The first null hypothesis was that repeated ceramic firings would not affect the marginal adaptation of metal-ceramic crowns. The second null hypothesis was that different fabrication methods would not significantly affect the marginal adaptation of metal copings.

## Materials and methods

**Fig 1:**
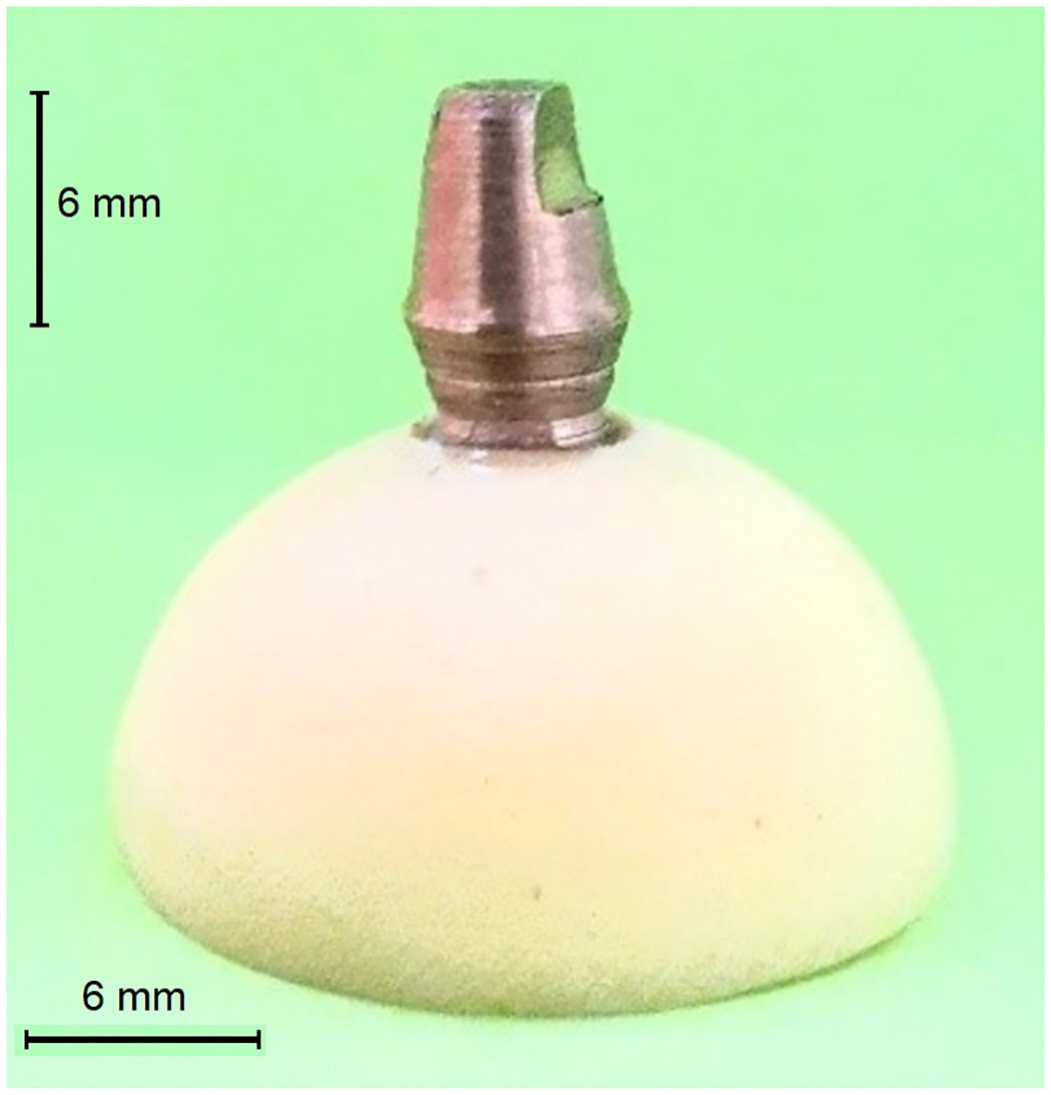
Brass master die.

A machined brass die was fabricated by using a computer numerical control lathe (CNC350; ARIX CNC Machines Co. Ltd, Taiwan) to simulate the preparation of a full coverage metal-ceramic restoration. Preparation was standardized with a 6-mm height, 6-mm width at the finish line, circumferential chamfer margin (0.7×2 mm [width×radius]), 6-degree convergence angle, and an anti-rotational surface. The die was placed in an autopolymerizing acrylic resin block (Crown & Bridge Resin; Dentsply Sirona, USA) and checked with a surveyor (Ney Dental Surveyor; Dentsply Sirona, USA) to ensure parallelism. This die was used to manufacture 20 copings (Fig 1).

Based on previous studies [25, 26] and by using a software, the specimens were equally divided into two groups of castable Ni-Cr (4-all; Ivoclar Vivadent; Schaan, Liechtenstein) and a new pre-sintered millabe Co-Cr (Ceramill Sintron, AmannGirrbach; Koblach, Austria), which could be processed by a desktop milling machine. This study assessed the marginal adaptation of specimens after 3, 5, and 7 porcelain firing cycles based on previous studies [3, 27], as clinically, the porcelains undergo a minimum of three firing cycles [28].

The master die was sprayed with a scan spray (Anti-Scan CAD-CAM Spray; Dr. Jean Bausch GmbH & Co KG, Germany) and scanned by using a 3D laser scanner (D810; 3Shape, Denmark). The scanned data were transferred to the CAD software (CAD Design software; 3Shape, Denmark). A pattern thickness of 0.5 mm and a cement space of 40 μm were chosen for the occlusal and axial surfaces, with no cement space at the margin. The CAD design output in form of a standard tessellation language (STL) file, was used to fabricate 20 patterns.

Milled copings were designed by the CAD software (Remote DENTAL 2.0; imes-icore GmbH, Hamburg, Germany). To be processed, the pre-sintered blanks were dry-milled with a milling machine (imes-icore; CORiTEC 340i, Hamburg, Germany). The cores were sintered in an argon atmosphere at 1300 °C (Magma preheating furnace; Renfert GmbH Hamburg, Germany) based on the manufacturer’s instructions.

**Fig 2:**
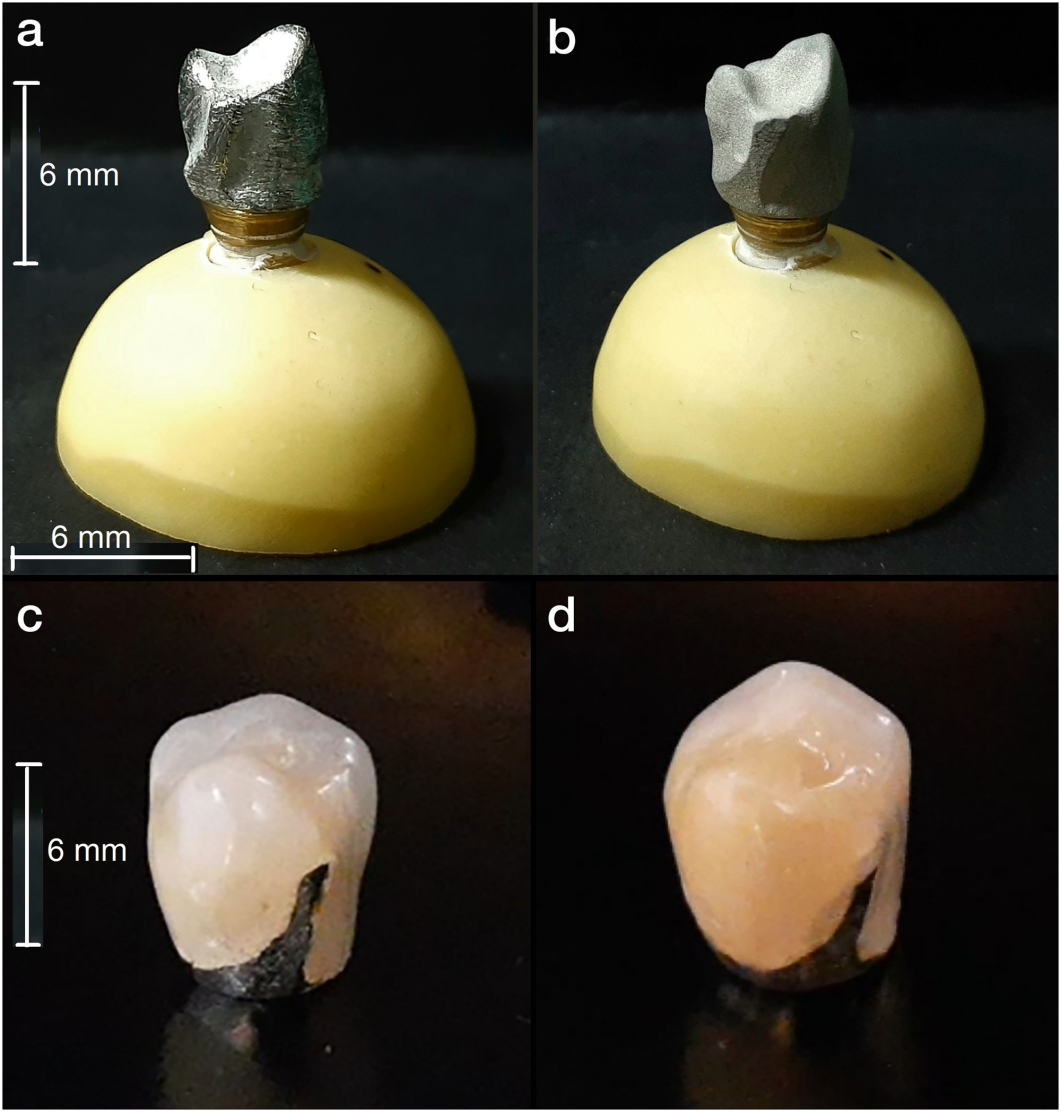
a: Cast coping, b: Milled coping, c: porcelain-fused-to-metal (PFM) crown fabricated by cast coping, d: PFM crown fabricated by milled coping.

In order to prepare the cast copings, wax patterns were fabricated from a CAD-CAM wax (Ceramill mall; Amann Girrbach, Austria) and cast (BEGO’s casting machines: Nautilus^®^ CC plus; USA) (Fig 2).

The copings were meticulously inspected before the application of veneering ceramic. Any defect on the intaglio surfaces that might obstruct the perfect seating of the copings was removed with a low-speed rotary instrument. The cameo surfaces were airborne-particle abraded with 110-μm aluminum oxide particles (Basic Classic; Renfert GmbH, Germany) for 10 seconds at approximately 0.2 MPa pressure from about 20-mm distance, based on a previous study [3]. The copings were then cleaned ultrasonically in 95% methyl alcohol for 15 minutes and dried using oil-free compressed air (Elmasonic S100H; Elma GmbH & Co KG, Germany).

An oxidized layer was obtained by placing the copings in the ceramic furnace (Programat EP 5000/G2; Ivoclar Vivadent AG, Liechtenstein). The opaque ceramic and veneering ceramic (IPS InLine; Ivoclar Vivadent AG, Liechtenstein) were applied according to the manufacturer’s instructions using a custom-made silicone mold to ensure a standardized ceramic thickness. Repeated ceramic firing cycles were performed in a dental ceramic furnace (Programat EP 5000/G2; Ivoclar Vivadent AG, Liechtenstein) to simulate clinical use. The initial four firings represented oxidation firing, opaque firing, dentin firing, and laboratory correction firing. The subsequent 3 firings represented the first and second correction firings and the glaze firing [1].

Once each restoration was seated on the master die, the assembly was mounted on a holding device. The restorations were identically positioned by the cone-shaped tip of the holding device, with a uniform load of 15 N [29]. The measurements were performed by a researcher who did not participate in the experimental stages.

**Fig 3:**
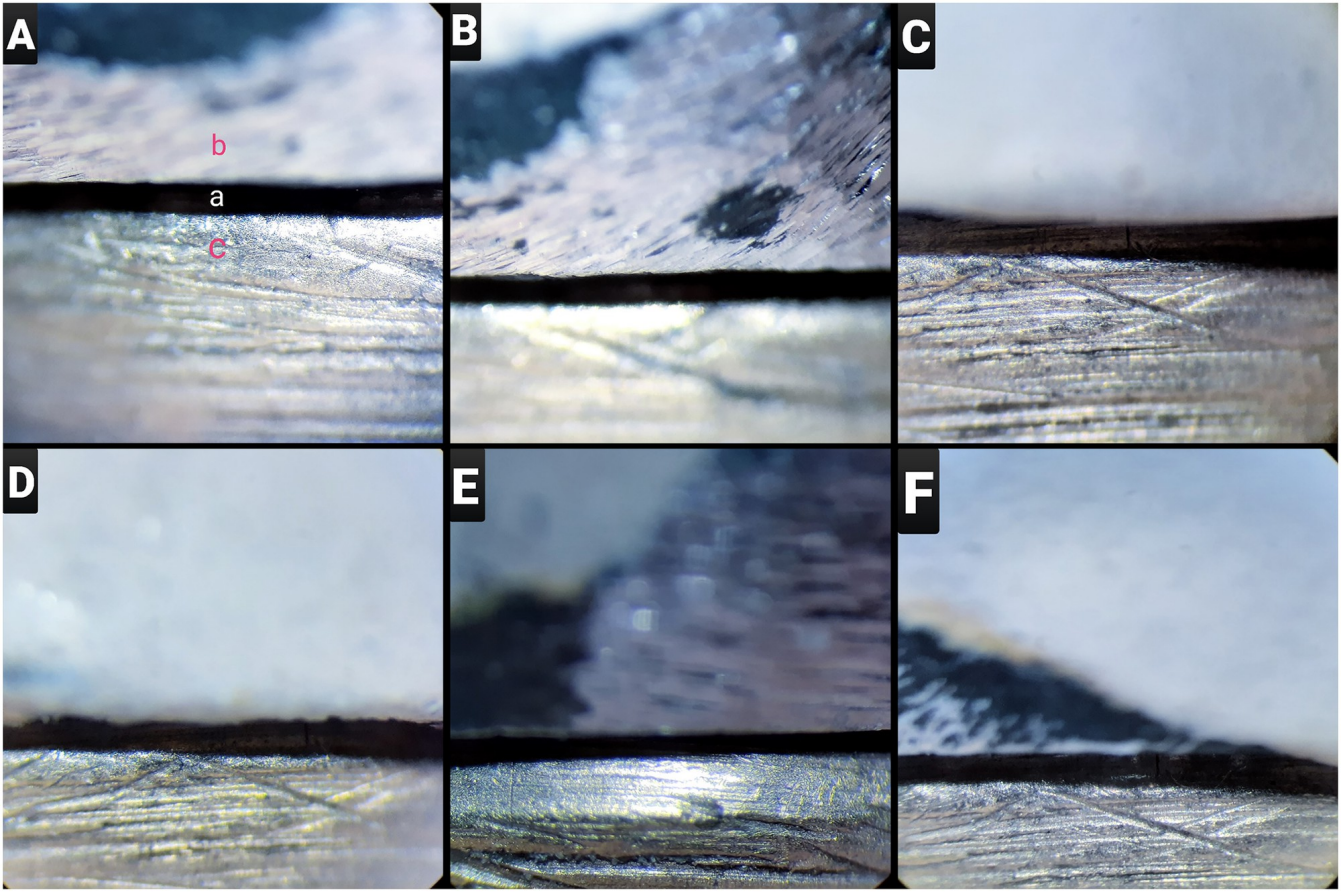
Microscopic image of PFM-die interface at 80× magnification; A. 4-all after 3 firing cycles (a: vertical marginal gap, b: PFM, c: brass die), B. 4-all after 5 firing cycles, C: 4-all after 7 firing cycles, D: Ceramill Sintron after 3 firing cycles, E: Ceramill Sintron after 5 firing cycles, F: Ceramill Sintron after 7 firing cycles.

To measure the marginal fit, 16 points (4 in each of the mesial, buccal, distal, and lingual) were marked and images at ×80 magnification were taken from each point by using a digital microscope (AM413FIT Dino-Lite Pro; AnMo Electronics Corp, Taiwan). The images were analyzed via an image analysis software (DinoCapture 2.0; AnMo Electronics Corp, Taiwan) and vertical misfits in each group were identified (Fig 3).

Statistical analyses of data were done through SPSS software (SPSS Inc; version 20, Chicago, Illinois, USA). The mean values and standard deviations were calculated in each group. Shapiro-Wilk test was used to evaluate the normal distribution, and the Levene test was used to assess the equality of variances. Repeated measures ANOVA and independent t-test were used to compare the study groups (*α* = 0.05).

## Results

**Fig 4:**
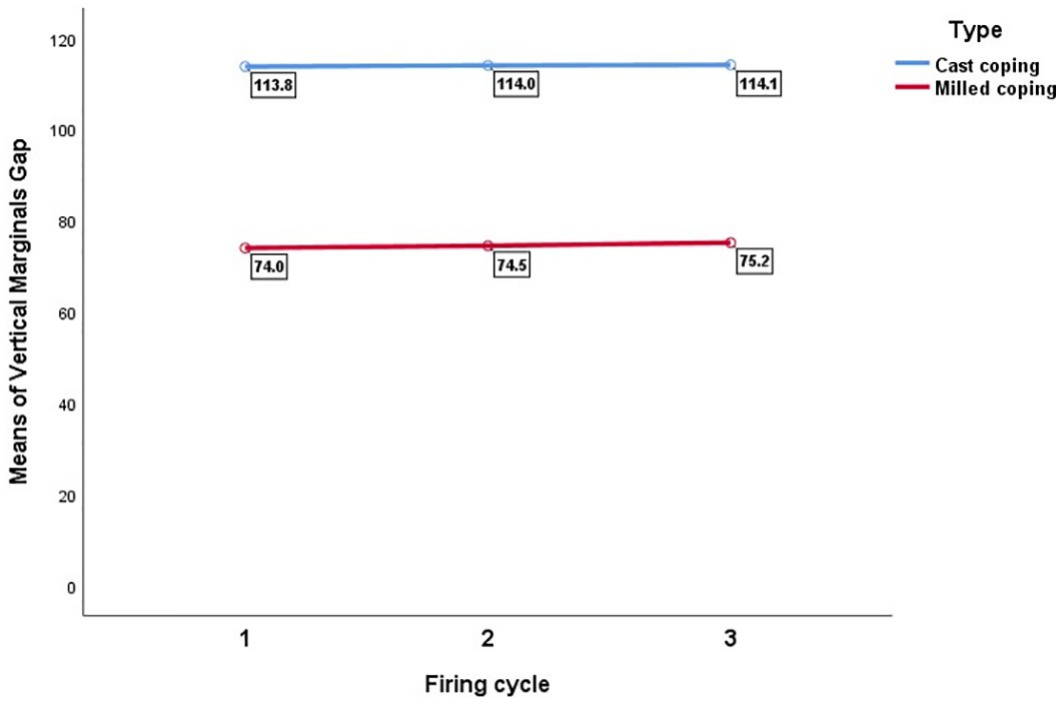
The mean vertical marginal gap (μm) of the two metal groups undergoing 3, 5, and 7 firing cycles.

Table 1 displays the means and standard deviations of the vertical marginal gap in both metal groups based on the number of firing cycles. The repeated measures two-way ANOVA showed no interaction between the repeated ceramic firing and fabrication technique (Table 2). The number of porcelain firing cycles had no significant effect on the marginal fit of any of the metal groups (*P* = 0.747). However, the marginal discrepancy of the milled copings was significantly lower than the cast copings (*P*<0.001) (Fig 4, Table 2).

**Table 1:** The mean and standard deviations of the vertical marginal gap of the two metal groups undergoing different firing cycles (μm).

Metal group	Firing cycles			P value	Reliability
	3	5	7		
Cast coping	113.8±8.8	114.0±6.5	114.1±4.5	0.980	ICC (95% CI)
Milled coping	74.0±6.1	74.5±7.27	75.2±6.9	0.377	0.85 (0.65–0.96)
P value	<0.001	<0.001	<0.001	----	0.97 (0.92–0.99)

Data were presented as mean ± SD, ICC: Intraclass Correlation Coefficient, CI: Confidence Interval

**Table 2:** Repeated measures two-way ANOVA for marginal gap evaluation.

Factor	Type III sum of square	df	Mean Square	F	P value	Partial Eta Squared
Repeated ceramic firing	5.84	2	2.94	0.294	0.747	0.016
Fabrication method	23320.9	1	23320.9	192.9	<0.001	0.915
Repeated ceramic firing × Fabrication method	1.83	2	0.901	0.091	0.914	0.005

**Fig 5:**
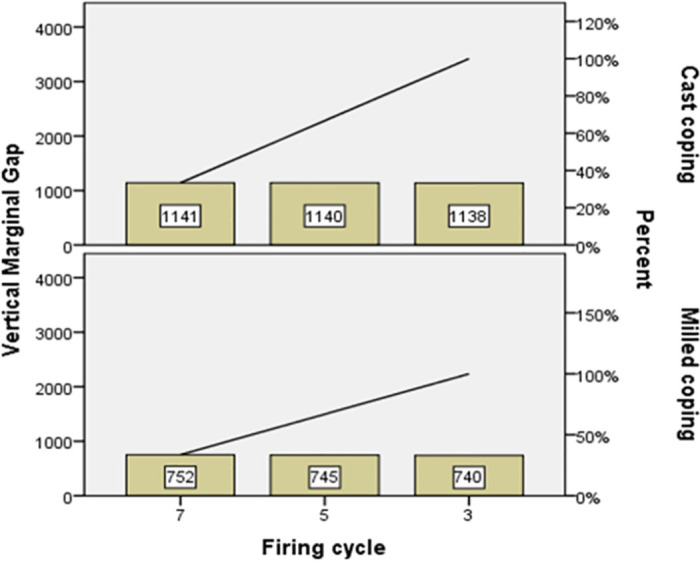
Pareto curve of the sum of the vertical marginal gap of the two metal groups undergoing different firing cycles.

**Fig 6:**
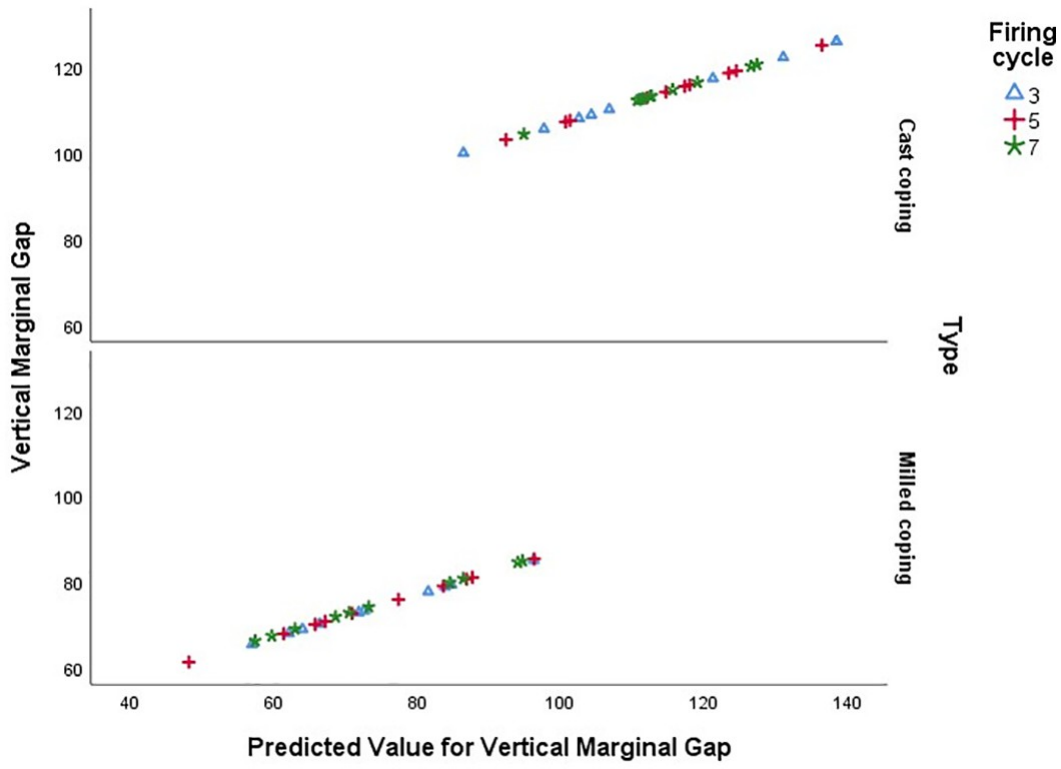
Scatter plot of input vertical marginal gap versus predicted value (output).

According to Table 1, there was no significant difference between the three numbers of firing cycles for the cast and milled coping metal types (*P* = 0.980 and *P* = 0.337 respectively). In addition, the intraclass correlation coefficient (ICC) indicated the internal consistency (Table 1). The Pareto curve was provided according to the sum of the vertical marginal gaps of the two metal groups undergoing different firing cycles that confirmed the above results (Fig 5, Table 3). To analyze the sensitivity, the scatter plot of the vertical marginal gap input value against its predicted value was plotted (Fig 6). This figure confirmed the absence of serious deviation between the input and output values of the vertical marginal gap.

**Table 3:** Regression model analysis results of Vertical marginal gap as a dependent variable.

Vertical marginal gap as a dependent variable	Parameter	Coefficient	SE of Coefficient	P value	Partial Eta Squared as effect size (ES)
Firing cycle 3	Intercept	74.0	2.41	<0.001	0.981
	Cast coping	39.78	3.14	<0.001	0.883
	Milled coping	Reference			
Firing cycle 5	Intercept	74.49	2.18	<0.001	0.985
	Cast coping	39.54	3.09	<0.001	0.901
	Milled coping	Reference			
Firing cycle 7	Intercept	75.18	1.85	<0.001	0.989
	Cast coping	39.96	2.62	<0.001	0.924
	Milled coping	Reference			

R^2^ of the regression model was 0.89

## Discussion

The first null hypothesis was accepted since repeated ceramic firing cycles had no significant effect on the marginal fit of the two metal groups. However, the second null hypothesis was rejected due to the significantly different marginal fits of the two metal groups after multiple firings.

### Repeated porcelain firings and marginal fit

Based on the present findings, the number of porcelain firing cycles did not affect the marginal fit of any of the metal groups, which was in line with Kocaağaoğlu et al.’s findings [1]. Similarly, Zeng et al. [8] observed that increasing the number of firing cycles did not significantly change the marginal fit of the copings made through selective laser melting and casting. In contrast, Önöral et al. [2] found that increasing the firing cycles to 2, 4, and 7 significantly affected the discrepancy values of frameworks fabricated through fully-sintered hard alloy milling, pre-sintered soft alloy milling, and selective laser sintering, but not those made through conventional casting. Likewise, Kaleli and Saraç [24] detected that the marginal discrepancy increased after ceramic firing.

Along with ceramic veneering, the metal frameworks undergo repeated firings at high temperatures, which distorts the metallurgical structure of the alloy and may adversely affect the fitting accuracy of metal-ceramic restorations [8]. Different factors contribute to this structural deformation such as the alloy type, ceramic shrinkage during firing, different thermal expansion coefficients of ceramic and metal, and residual stress on the frameworks originating from the stages before firing and creep of the alloys at high temperatures [1, 8]. In the current study, the marginal fit of neither group was significantly affected by repeated firing cycles. There is a close relationship between the marginal accuracy and the integrity of the margins of the metal coping. Thus, changes in the metallurgical phases of the alloy during porcelain firing cannot directly affect the marginal accuracy of the metal coping, because repeated porcelain firing does not necessarily destroy the margin of the metal coping [8].

### Type of framework and marginal fit

Based on the current findings, the mean vertical marginal gap of the millable alloy was significantly lower than the vertical marginal gap of the cast alloy, which could significantly contribute to the long-term survival of metal-ceramic restorations. In line with the present study, Kocaağaoğlu et al. [1] detected that the copings made via laser sintering and soft milling had a better marginal fit than those made through the lost wax technique. Önöral et al. [2] evaluated the marginal fit of 3-unit fixed partial dentures manufactured with different framework methods including casting, fully-sintered hard alloy milling, pre-sintered soft alloy milling, and selective laser sintering. The most accurate fitting was seen in the restorations made through pre-sintered soft alloy milling. In both studies, the higher marginal discrepancy of copings fabricated by casting was attributed to the challenging nature and sensitivity of the casting method.

In contrast, milling does not require duplication or trimming of a stone cast; and making a crown pattern only takes 7 minutes. These improvements reduce the turnaround time and errors in the manufacturing process [30]. Additionally, casting procedure exposes a compressive load (450 MPa) on the alloy, the extra amount of which are released during the firing and can negatively affect the fit of a cast restoration [31]. Seemingly, casting and melting an alloy may increase the marginal discrepancy.

In the present study, standardization of metal copings and application of veneering ceramic were all done by the same skilled dental technician. The metal copings were all made based on the same design with standardized thickness; a custom-made silicone mold was also used to standardize the veneering ceramic thickness.

In the present study, a single brass die was employed to standardize the preparation and avoid any wear throughout the seating and measuring process. Measurements of the two groups were performed on this single die. Moreover, not cementing the copings on the master die precluded the interfering factors such as the type and viscosity of the luting agent and seating forces during cementation [24, 29]. A holding device was used to standardize the seating of the copings on the die during measurements.

The marginal fit of metal-ceramic restorations has been evaluated through different methods including direct microscopic imaging [29], microscopic measurement of the cement thickness [32], examination of the intaglio of dental restorations via micro-computed tomography [33], 3D surface scanning [7], and silicone replica technique [2, 8]. Direct measurement of the cement thickness offers more precise observations since the cross-section can be directly checked. However, this method destroys the copings and brass die, hindering repeated measurements on the same specimen. Micro-computed tomography and 3D surface scanning are costly and require advanced technologies. Extra measurement errors might accompany the replica technique because the silicone film is placed between the prosthetic element and the die. Such errors are attributed to the silicone viscosity, complicated determination of the coping margins and die finish line, and peeling off and tearing of the thin layer of elastomeric material when removing from the die [34].

In the present study, the marginal discrepancy of metal copings was measured through a direct microscopic view, which is a commonly-used nondestructive method for reproducible results that measures the marginal discrepancy in unlimited points [29, 30]. Moreover, it allows direct visualization and gauging of the vertical distance between the finish line of the die and the margin of the copings with no need for cement, embedment resin, or sectioning of specimens. This technique is more economical, less time-consuming, and more precise than other extensive and multiple-step methods. However, the direct microscopic view does not allow the assessment of the horizontal marginal gap [29, 30].

There is no universally accepted value for the marginal accuracy of restorations. Fransson et al. [35] reported that the marginal discrepancy value of the restorations should theoretically be between 25 and 50 μm; however, this value is clinically difficult to achieve. McLean and von Fraunhofer [22] reported that the maximum clinically acceptable marginal discrepancy should be 120 μm for the long-term success of restorations. In the present study, all groups had marginal discrepancy values of less than 120 μm at all firing stages, which is clinically acceptable. This finding suggests that restorations made by using either of the two studied fabrication techniques are suitable for clinical use; however, more studies, including clinical trials, are required.

The finish-line configuration has been reported to influence the fitting accuracy of restorations [21]. In a recent systematic review and meta-analysis of finish line preparations of Co-Cr copings made via CAD-CAM, the overall marginal and internal adaptation was found to be better with shoulder and rounded shoulder finish line designs when the copings were fabricated through CAD-CAM methods. Soft milling and direct metal laser sintering created better marginal and internal adaptation in chamfer and deep chamfer finish line designs; however, hard milling presented better shoulder finish line [36]. The chamfer finish line has been used in the present study to evaluate the adaptation of porcelain-fused-to-metal crowns.

Studies have achieved different results most probably due to the differences in specimen preparation, testing methodology, methods used to evaluate fitting accuracy, sample size, number of measurement points, research type (*in-vivo* versus *in-vitro*), employed materials, and the use of software, all of which can influence the fitting accuracy of restorations. It might also be attributed to the wax pattern deformation, precision of CAD-CAM systems, and the impression material shrinkage [1, 2].

Among the several limitations of this study was evaluating only three steps of firing cycles. Future studies are suggested to assess even greater numbers of firing cycles. Moreover, the advanced technology of 3D imaging was not available to be used for measurements; nor was the selective laser melting technique included. Furthermore, the *in-vitro* design could not accurately reflect the clinical situation.

## Conclusions

Within the limitations of this study, it can be concluded that:

1. Repeated porcelain firing cycles have no significant effect on the marginal fit of casting and millable alloys.
2. The millable alloy has superior marginal fit accuracy regardless of the number of porcelain firing cycles.
3. Repeated firings do not increase the marginal fit of the studied groups beyond the clinically acceptable range (<120 μm).
4. The millable Co-Cr alloy is an alternative to the casting alloy for metal-ceramic restorations and CAD-CAM can be efficiently used in porcelain-fused-to-metal restorations.
